# Crystal structure of chlorido­[1-(4-nitro­phen­yl)thio­urea-κ*S*]bis­(tri­phenyl­phosphane-κ*P*)copper(I)

**DOI:** 10.1107/S2056989016019368

**Published:** 2017-01-01

**Authors:** Arunpatcha Nimthong-Roldán, Nichakan Promsuwhan, Walailak Puetpaiboon, Yupa Wattanakanjana

**Affiliations:** a Department of Chemistry, Boston University, Boston, Massachusetts 02215, USA; b Department of Chemistry, Faculty of Science, Prince of Songkla University, Hat Yai, Songkhla 90112, Thailand

**Keywords:** crystal structure, N—H⋯Cl hydrogen bonding, intra- and inter­molecular hydrogen bonding

## Abstract

The mononuclear mixed-ligand title complex, [CuCl(C_7_H_7_N_3_O_2_S)(C_18_H_15_P)_2_], displays a distorted tetra­hedral coordination sphere around the Cu^I^ atom, with two P atoms from two tri­phenyl­phosphane mol­ecules, one terminal S atom from a 1-(4-nitro­phen­yl)thio­urea mol­ecule and a chloride ion as ligands. An intra­molecular N—H⋯Cl hydrogen bond stabilizes the mol­ecular conformation [graph-set motif *R*_2_^2^(6)]. In the crystal, further N—H⋯Cl hydrogen bonds connect individual mol­ecules into zigzag chains parallel to [001]. The chains are linked by weak C—H⋯O hydrogen-bonding inter­actions into a three-dimensional network.

## Abstract 2

The mononuclear title complex contains a chloride, a 1-(4-nitro­phen­yl)thio­urea and two tri­phenyl­phosphane ligands, leading to a tetra­hedrally arranged ClP_2_S coordination set. N—H⋯Cl and C—H⋯O hydrogen bonds connect the mol­ecules into a three-dimensional network.

## Chemical context   

Thio­urea and thio­urea derivatives constitute an inter­esting class of ligands, bearing a soft sulfur and a hard nitro­gen donor atom in the sense of the HSAB (hard and soft acids and bases) concept. Such ligands are of relevance in biological systems because they exhibit a moderate inhibitory potency on the diphenolase activity of tyrosinase (Liu *et al.*, 2016 ▸), anti­microbial and cytotoxic activity (Bielenica *et al.*, 2015 ▸) and are developed for anti-hepatitis C virus (HCV) activity (Khatri *et al.*, 2015 ▸). Copper(I) complexes with thio­urea derivatives have received significant attention for several decades due to their anti­bacterial activity (Chetana *et al.*, 2016 ▸), cytotoxic activity (Rauf *et al.*, 2009 ▸), catalytic and oxidation properties (Gunasekaran *et al.*, 2017 ▸). In this context, we report here on synthesis and crystal structure of the title compound, [CuCl(C_7_H_7_N_3_O_2_S)(C_18_H_15_P)_2_], (I).
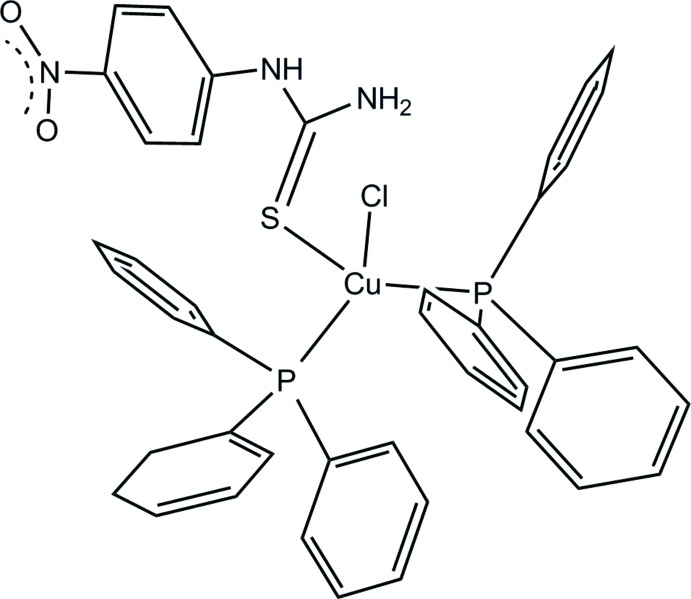


## Structural commentary   

**Figure 1:**
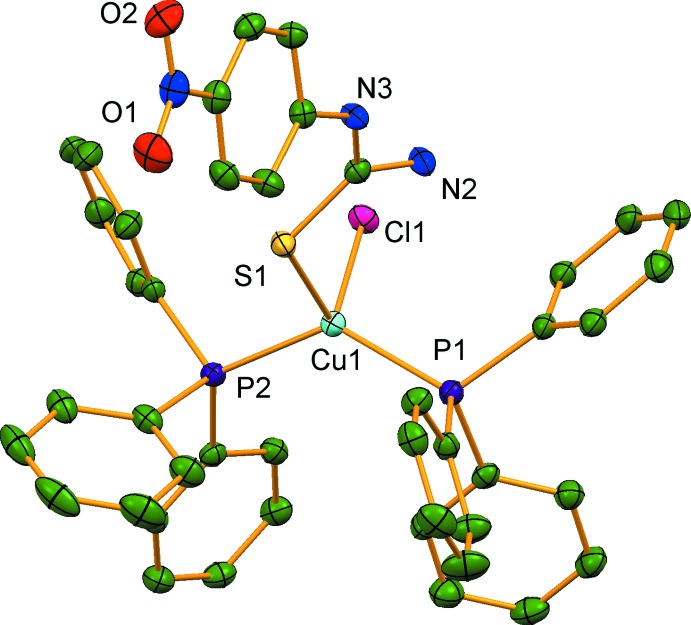
The mol­ecular structure of (I), with displacement ellipsoids drawn at the 50% probability level. All H atoms have been omitted for clarity.

The asymmetric unit of (I) comprises of one Cu^I^ atom, one chloride ligand, two tri­phenyl­phosphane (PPh_3_) ligands, and one 1-(4-nitro­phen­yl)thio­urea (NPTU) ligand. The distorted tetra­hedral coordination of the Cu^I^ atom results from binding to the chloride ligand, the P atoms of the two PPh_3_ ligands and the terminal S atom of the 1-(4-nitro­phen­yl)thio­urea ligand (Fig. 1 ▸). The distortion is evident from the angular range around the Cu^I^ atom [99.870 (15)–129.119 (16)°] and the disparate bond lengths (Table 1 ▸). The Cu—S distance in (I) is somewhat smaller than the values of 2.4148 (16) and 2.3942 (15) Å reported in mol­ecules *A* and *B*, respectively, of [CuI(PPh_3_)_2_(ptu)] (ptu is phenyl thio­urea) (Nimthong *et al.*, 2008 ▸). The formation of an intra­molecular N—H⋯Cl hydrogen bond involving the primary amine functionality (N2—H2*B*; Table 2 ▸) creates a six-membered ring system with graph set motif 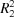
(6).

## Supra­molecular features   

**Figure 2:**
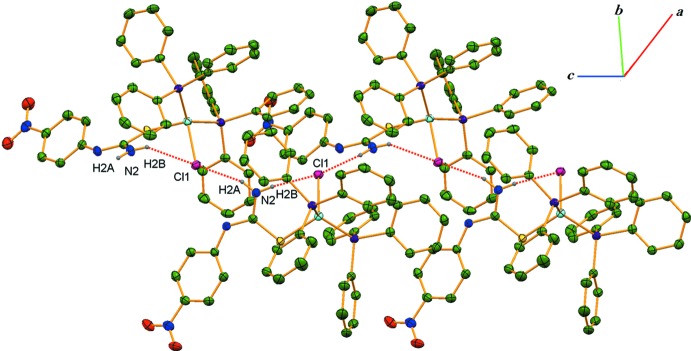
Part of the crystal structure of (I), showing inter­molecular N—H⋯Cl hydrogen bonds as dashed lines, forming a zigzag chain parallel to [001].

**Figure 3:**
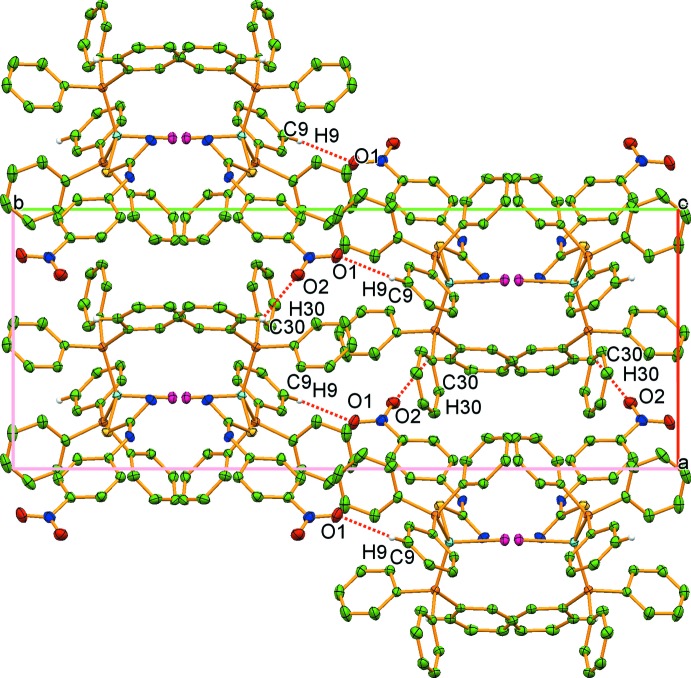
Part of the crystal structure of (I), showing the three-dimensional network formed by inter­molecular C—H⋯O hydrogen bonds (shown as dashed lines).

In the crystal, neighbouring mol­ecules are linked by further N—H⋯Cl hydrogen bonds between the NPTU NH2 (N2—H2*A*) and NHPh (N1—H3*A*) moieties and the chloride ligands into zigzag chains extending parallel to [001] (Fig. 2 ▸, Table 2 ▸). The chains are connected *via* weak C9—H9⋯O1 and C30—H30⋯O2 hydrogen bonds (Fig. 3 ▸, Table 2 ▸), leading to the formation of a three-dimensional network (Fig. 3 ▸).

## Database survey   

A search of the Cambridge Structural Database (Version 5.37, Feb 2016 with two updates; Groom *et al.*, 2016 ▸) revealed no complexes with the 1-(4-nitro­phen­yl)thio­urea ligand, and only the crystal structure of the ligand itself has been reported (LONSEN; Xian *et al.*, 2008 ▸). A search for phenyl­thio­urea ligands with substitutions on the phenyl ring yielded 34 hits. Of these, four hits were Cu^I^ complexes, namely IYUXOP01 (Li *et al.*, 2006 ▸), TULXIJ, TULXUV (Grifasi *et al.*, 2015 ▸) and TULXUV (Nimthong *et al.*, 2008 ▸).

## Synthesis and crystallization   

Tri­phenyl­phosphane (0.26 g, 0.99 mmol) was dissolved in 30 ml of aceto­nitrile at 338 K and then copper(I) chloride (0.1 g, 1.01 mmol) was added. The mixture was stirred for 3 h and then 1-(4-nitro­phen­yl)thio­urea, (0.2 g, 1.01 mmol) was added. The resulting reaction mixture was heated under reflux for 3 h during which the precipitate gradually disappeared. The resulting clear solution was filtered and left to evaporate at room temperature. The crystalline complex, which deposited upon standing for a couple of days, was filtered off and dried *in vacuo* (0.38 g, 45% yield). M.p. 483–485 K. IR bands (KBr, cm^−1^): 3066 (*m*), 3049 (*m*), 3017 (*m*), 2345 (*w*), 1961 (*w*), 1890 (*w*), 1814 (*w*), 1582 (*w*), 1474 (*s*), 1433 (*s*), 1307 (*w*), 1268 (*w*), 1176 (*m*), 1153 (*m*), 1088 (s), 1065 (*m*), 1024 (*s*), 994 (*m*), 916 (*w*), 852 (*m*), 741 (*s*), 692 (*s*).

## Refinement   

Crystal data, data collection and structure refinement details are summarized in Table 3 ▸. H atoms attached to carbon atoms were positioned geometrically and constrained to ride on their parent atoms, with C—H = 0.95 Å. Nitro­gen-bound H atoms were located in difference density maps and were refined with an N—H distance restraint of 0.88 (2) Å. *U*_iso_(H) values were set to 1.2*U*_eq_(C/N).

## Supplementary Material

Crystal structure: contains datablock(s) I. DOI: 10.1107/S2056989016019368/wm5344sup1.cif

Structure factors: contains datablock(s) I. DOI: 10.1107/S2056989016019368/wm5344Isup2.hkl

CCDC reference: 1520741

Additional supporting information: crystallographic information; 3D view; checkCIF report

## Figures and Tables

**Table 1:** Selected geometric parameters (Å, °)

Cu1—P2	2.2602 (4)	Cu1—S1	2.3782 (4)
Cu1—P1	2.2671 (4)	Cu1—Cl1	2.4023 (4)
			
P2—Cu1—P1	129.119 (16)	P2—Cu1—Cl1	99.870 (15)
P2—Cu1—S1	101.267 (15)	P1—Cu1—Cl1	109.823 (16)
P1—Cu1—S1	110.861 (15)	S1—Cu1—Cl1	102.637 (15)

**Table 2:** Hydrogen-bond geometry (Å, °)

*D*—H⋯*A*	*D*—H	H⋯*A*	*D*⋯*A*	*D*—H⋯*A*
N2—H2*A*⋯Cl1^i^	0.88 (2)	2.35 (2)	3.1974 (14)	160 (2)
N2—H2*B*⋯Cl1	0.88 (1)	2.42 (2)	3.2504 (15)	158 (2)
N3—H3*A*⋯Cl1^i^	0.87 (1)	2.49 (2)	3.3199 (14)	158 (2)
C9—H9⋯O1^ii^	0.95	2.57	3.303 (2)	135
C30—H30⋯O2^iii^	0.95	2.70	3.386 (2)	130

Symmetry codes: (i) 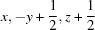; (ii) 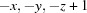; (iii) 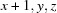.

**Table 3:** Experimental details

Crystal data	
Chemical formula	[CuCl(C_7_H_7_N_3_O_2_S)(C_18_H_15_P)_2_]
*M*_r_	820.74
Crystal system, space group	Monoclinic, *P*2_1_/*c*
Temperature (K)	100
*a*, *b*, *c* (Å)	11.6986 (1), 28.7847 (4), 11.8471 (1)
β (°)	106.3394 (9)
*V* (Å^3^)	3828.28 (7)
*Z*	4
Radiation type	Mo *K*α
μ (mm^−1^)	0.82
Crystal size (mm)	0.45 × 0.32 × 0.20

Data collection	
Diffractometer	Nonius KappaCCD
Absorption correction	Multi-scan (*SCALEPACK*; Otwinowski & Minor, 1997 ▸)
*T*_min_, *T*_max_	0.746, 0.853
No. of measured, independent and observed [*I* > 2σ(*I*)] reflections	37561, 10435, 8243
*R*_int_	0.034
(sin θ/λ)_max_ (Å^−1^)	0.720

Refinement	
*R*[*F* ^2^ > 2σ(*F* ^2^)], *wR*(*F* ^2^), *S*	0.035, 0.095, 1.10
No. of reflections	10435
No. of parameters	488
No. of restraints	3
H-atom treatment	H atoms treated by a mixture of independent and constrained refinement
Δρ_max_, Δρ_min_ (e Å^−3^)	0.53, −0.70

Computer programs: *COLLECT* (Nonius, 1998 ▸), *HKL-3000* (Otwinowski & Minor, 1997 ▸), *SHELXS97* (Sheldrick, 2008 ▸), *SHELXL2014* (Sheldrick, 2015 ▸), *SHELXLE* (Hübschle *et al.*, 2011 ▸), *Mercury* (Macrae *et al.*, 2008 ▸) and *publCIF* (Westrip, 2010 ▸).

## supplementary crystallographic information

### Crystal data

**Table:**

[CuCl(C_7_H_7_N_3_O_2_S)(C_18_H_15_P)_2_]	*F*(000) = 1696
*M**_r_* = 820.74	*D*_x_ = 1.424 Mg m^−^^3^
Monoclinic, *P*2_1_/*c*	Mo *K*α radiation, λ = 0.71073 Å
*a* = 11.6986 (1) Å	Cell parameters from 37561 reflections
*b* = 28.7847 (4) Å	θ = 1.9–30.8°
*c* = 11.8471 (1) Å	µ = 0.82 mm^−^^1^
β = 106.3394 (9)°	*T* = 100 K
*V* = 3828.28 (7) Å^3^	Plate, yellow
*Z* = 4	0.45 × 0.32 × 0.20 mm

### Data collection

**Table:**

Nonius KappaCCD diffractometer	10435 independent reflections
Radiation source: fine focus X-ray tube	8243 reflections with *I* > 2σ(*I*)
Graphite monochromator	*R*_int_ = 0.034
ω and φ scans	θ_max_ = 30.8°, θ_min_ = 1.9°
Absorption correction: multi-scan (*SCALEPACK*; Otwinowski & Minor, 1997)	*h* = −15→16
*T*_min_ = 0.746, *T*_max_ = 0.853	*k* = −31→38
37561 measured reflections	*l* = −15→12

### Refinement

**Table:**

Refinement on *F*^2^	Secondary atom site location: difference Fourier map
Least-squares matrix: full	Hydrogen site location: inferred from neighbouring sites
*R*[*F*^2^ > 2σ(*F*^2^)] = 0.035	H atoms treated by a mixture of independent and constrained refinement
*wR*(*F*^2^) = 0.095	*w* = 1/[σ^2^(*F*_o_^2^) + (0.0511*P*)^2^ + 0.4104*P*] where *P* = (*F*_o_^2^ + 2*F*_c_^2^)/3
*S* = 1.10	(Δ/σ)_max_ = 0.002
10435 reflections	Δρ_max_ = 0.53 e Å^−^^3^
488 parameters	Δρ_min_ = −0.70 e Å^−^^3^
3 restraints	Extinction correction: SHELXL2014 (Sheldrick, 2015), Fc^*^=kFc[1+0.001xFc^2^λ^3^/sin(2θ)]^-1/4^
Primary atom site location: structure-invariant direct methods	Extinction coefficient: 0.0016 (3)

### Special details

**Table:**

Geometry. All esds (except the esd in the dihedral angle between two l.s. planes) are estimated using the full covariance matrix. The cell esds are taken into account individually in the estimation of esds in distances, angles and torsion angles; correlations between esds in cell parameters are only used when they are defined by crystal symmetry. An approximate (isotropic) treatment of cell esds is used for estimating esds involving l.s. planes.

### Fractional atomic coordinates and isotropic or equivalent isotropic displacement parameters (Å^2^)

**Table:**

	*x*	*y*	*z*	*U*_iso_*/*U*_eq_	
Cu1	0.28282 (2)	0.15638 (2)	0.54942 (2)	0.01735 (6)	
O1	−0.17866 (13)	0.01265 (5)	0.98751 (12)	0.0385 (3)	
N1	−0.18661 (13)	0.05452 (6)	1.00307 (13)	0.0286 (3)	
S1	0.14096 (4)	0.14121 (2)	0.65448 (3)	0.01970 (9)	
Cl1	0.27367 (4)	0.23943 (2)	0.52868 (3)	0.02252 (9)	
P1	0.46802 (4)	0.13469 (2)	0.65603 (3)	0.01731 (9)	
C1	0.58276 (14)	0.13035 (6)	0.57889 (14)	0.0193 (3)	
P2	0.17975 (4)	0.13535 (2)	0.36507 (3)	0.01688 (9)	
N2	0.26531 (13)	0.20750 (5)	0.78940 (12)	0.0226 (3)	
H2A	0.2850 (17)	0.2247 (6)	0.8536 (14)	0.027*	
H2B	0.2894 (17)	0.2142 (7)	0.7271 (15)	0.027*	
O2	−0.25332 (12)	0.07159 (5)	1.05564 (12)	0.0357 (3)	
C2	0.70247 (15)	0.14023 (6)	0.63207 (15)	0.0242 (3)	
H2	0.7269	0.1515	0.7106	0.029*	
C3	0.78635 (16)	0.13360 (6)	0.57006 (17)	0.0284 (4)	
H3	0.8678	0.1406	0.6062	0.034*	
N3	0.12426 (13)	0.17546 (5)	0.86177 (12)	0.0205 (3)	
H3A	0.1444 (17)	0.1990 (6)	0.9099 (15)	0.025*	
C4	0.63253 (17)	0.10666 (7)	0.40297 (16)	0.0305 (4)	
H4	0.6086	0.0949	0.3249	0.037*	
C6	0.75117 (17)	0.11684 (6)	0.45616 (16)	0.0298 (4)	
H6	0.8086	0.1123	0.4142	0.036*	
C7	0.25157 (14)	0.13084 (5)	0.24718 (13)	0.0185 (3)	
C8	0.22295 (15)	0.09615 (6)	0.16151 (14)	0.0220 (3)	
H8	0.1626	0.0741	0.1620	0.026*	
C9	0.28264 (15)	0.09374 (6)	0.07539 (15)	0.0256 (4)	
H9	0.2639	0.0697	0.0181	0.031*	
C10	0.36928 (15)	0.12618 (6)	0.07284 (14)	0.0244 (3)	
H10	0.4096	0.1245	0.0137	0.029*	
C11	0.39707 (15)	0.16098 (6)	0.15629 (15)	0.0244 (4)	
H11	0.4555	0.1835	0.1536	0.029*	
C12	0.33950 (15)	0.16309 (6)	0.24439 (14)	0.0216 (3)	
H12	0.3603	0.1866	0.3028	0.026*	
C13	0.05888 (14)	0.17673 (5)	0.30754 (13)	0.0190 (3)	
C14	−0.02848 (15)	0.18109 (6)	0.36695 (14)	0.0252 (4)	
H14	−0.0266	0.1612	0.4315	0.030*	
C15	−0.11740 (16)	0.21401 (7)	0.33257 (16)	0.0301 (4)	
H15	−0.1775	0.2161	0.3721	0.036*	
C16	−0.11927 (16)	0.24407 (6)	0.24056 (15)	0.0292 (4)	
H16	−0.1803	0.2668	0.2173	0.035*	
C17	−0.03195 (16)	0.24083 (6)	0.18273 (15)	0.0272 (4)	
H17	−0.0324	0.2616	0.1203	0.033*	
C18	0.05679 (15)	0.20707 (6)	0.21616 (14)	0.0221 (3)	
H18	0.1163	0.2049	0.1759	0.027*	
C19	0.10921 (15)	0.07869 (6)	0.36250 (14)	0.0219 (3)	
C20	0.18067 (18)	0.04382 (6)	0.42833 (16)	0.0298 (4)	
H20	0.2598	0.0508	0.4730	0.036*	
C21	0.1372 (2)	−0.00109 (7)	0.42928 (18)	0.0397 (5)	
H21	0.1869	−0.0248	0.4727	0.048*	
C22	0.0206 (2)	−0.01094 (7)	0.36626 (17)	0.0409 (5)	
H22	−0.0099	−0.0415	0.3677	0.049*	
C23	−0.05114 (19)	0.02317 (7)	0.30175 (16)	0.0366 (5)	
H23	−0.1309	0.0161	0.2591	0.044*	
C24	−0.00719 (16)	0.06825 (7)	0.29855 (15)	0.0273 (4)	
H24	−0.0566	0.0916	0.2529	0.033*	
C25	0.53685 (14)	0.17298 (6)	0.78059 (13)	0.0189 (3)	
C26	0.54102 (14)	0.22062 (6)	0.75712 (15)	0.0228 (3)	
H26	0.5135	0.2316	0.6785	0.027*	
C27	0.58508 (15)	0.25178 (6)	0.84823 (16)	0.0272 (4)	
H27	0.5884	0.2840	0.8317	0.033*	
C28	0.62437 (15)	0.23607 (7)	0.96356 (16)	0.0291 (4)	
H28	0.6532	0.2575	1.0261	0.035*	
C29	0.62144 (16)	0.18898 (7)	0.98716 (15)	0.0283 (4)	
H29	0.6489	0.1782	1.0660	0.034*	
C30	0.57845 (15)	0.15744 (6)	0.89593 (14)	0.0229 (3)	
H30	0.5776	0.1252	0.9126	0.027*	
C31	0.47481 (14)	0.07718 (6)	0.72334 (13)	0.0202 (3)	
C32	0.39244 (15)	0.06663 (6)	0.78530 (14)	0.0236 (3)	
H32	0.3346	0.0891	0.7906	0.028*	
C33	0.39449 (16)	0.02369 (6)	0.83911 (14)	0.0264 (4)	
H33	0.3390	0.0171	0.8821	0.032*	
C34	0.47685 (18)	−0.00944 (7)	0.83038 (16)	0.0333 (4)	
H34	0.4779	−0.0389	0.8669	0.040*	
C35	0.5581 (2)	0.00040 (7)	0.76808 (19)	0.0397 (5)	
H35	0.6145	−0.0224	0.7616	0.048*	
C36	0.55731 (18)	0.04345 (7)	0.71507 (17)	0.0316 (4)	
H36	0.6135	0.0499	0.6728	0.038*	
C38	0.17955 (14)	0.17619 (6)	0.77521 (13)	0.0191 (3)	
C40	0.04170 (14)	0.14409 (6)	0.88460 (13)	0.0195 (3)	
C41	−0.03251 (15)	0.16167 (6)	0.94837 (14)	0.0225 (3)	
H41	−0.0313	0.1939	0.9654	0.027*	
C42	−0.10781 (15)	0.13253 (6)	0.98702 (14)	0.0244 (4)	
H42	−0.1569	0.1443	1.0321	0.029*	
C43	−0.11000 (15)	0.08595 (6)	0.95871 (14)	0.0237 (3)	
C44	−0.03983 (16)	0.06778 (6)	0.89303 (15)	0.0257 (4)	
H44	−0.0438	0.0357	0.8736	0.031*	
C45	0.03635 (16)	0.09711 (6)	0.85594 (14)	0.0245 (3)	
H45	0.0851	0.0851	0.8108	0.029*	
C5	0.54875 (15)	0.11370 (6)	0.46403 (15)	0.0261 (4)	
H5	0.4672	0.1071	0.4270	0.031*	

### Atomic displacement parameters (Å^2^)

**Table:**

	*U*^11^	*U*^22^	*U*^33^	*U*^12^	*U*^13^	*U*^23^
Cu1	0.01643 (11)	0.01770 (11)	0.01733 (10)	0.00078 (7)	0.00379 (7)	−0.00070 (7)
O1	0.0449 (9)	0.0264 (8)	0.0473 (8)	−0.0121 (6)	0.0183 (7)	0.0012 (6)
N1	0.0257 (8)	0.0294 (9)	0.0295 (8)	−0.0044 (6)	0.0058 (6)	0.0061 (6)
S1	0.0188 (2)	0.0219 (2)	0.01894 (18)	−0.00284 (15)	0.00621 (14)	−0.00332 (14)
Cl1	0.0282 (2)	0.01691 (19)	0.02100 (18)	0.00003 (15)	0.00450 (15)	0.00155 (14)
P1	0.0162 (2)	0.0165 (2)	0.01874 (19)	0.00010 (15)	0.00413 (15)	0.00061 (14)
C1	0.0185 (8)	0.0165 (8)	0.0230 (8)	0.0021 (6)	0.0059 (6)	0.0031 (6)
P2	0.0168 (2)	0.0162 (2)	0.01734 (19)	−0.00007 (15)	0.00429 (14)	−0.00065 (14)
N2	0.0227 (7)	0.0255 (8)	0.0204 (7)	−0.0064 (6)	0.0075 (6)	−0.0053 (6)
O2	0.0300 (7)	0.0390 (8)	0.0435 (8)	−0.0004 (6)	0.0192 (6)	0.0092 (6)
C2	0.0221 (9)	0.0226 (9)	0.0285 (8)	−0.0008 (7)	0.0082 (7)	−0.0006 (7)
C3	0.0198 (9)	0.0265 (10)	0.0406 (10)	−0.0020 (7)	0.0114 (7)	0.0005 (7)
N3	0.0241 (7)	0.0187 (7)	0.0205 (6)	−0.0042 (6)	0.0092 (5)	−0.0037 (5)
C4	0.0322 (10)	0.0332 (10)	0.0278 (9)	0.0032 (8)	0.0110 (7)	−0.0021 (7)
C6	0.0296 (10)	0.0261 (9)	0.0395 (10)	0.0042 (8)	0.0191 (8)	0.0035 (8)
C7	0.0177 (8)	0.0185 (8)	0.0189 (7)	0.0037 (6)	0.0044 (6)	0.0020 (6)
C8	0.0216 (8)	0.0207 (8)	0.0236 (8)	−0.0011 (6)	0.0062 (6)	−0.0013 (6)
C9	0.0275 (9)	0.0255 (9)	0.0245 (8)	0.0007 (7)	0.0086 (7)	−0.0041 (7)
C10	0.0257 (9)	0.0262 (9)	0.0238 (8)	0.0059 (7)	0.0113 (7)	0.0031 (6)
C11	0.0245 (9)	0.0200 (9)	0.0310 (9)	−0.0005 (7)	0.0119 (7)	0.0026 (6)
C12	0.0219 (8)	0.0184 (8)	0.0248 (8)	−0.0002 (6)	0.0069 (6)	−0.0016 (6)
C13	0.0173 (8)	0.0170 (8)	0.0202 (7)	−0.0005 (6)	0.0010 (6)	−0.0032 (6)
C14	0.0198 (8)	0.0304 (10)	0.0257 (8)	0.0027 (7)	0.0066 (6)	0.0010 (7)
C15	0.0203 (9)	0.0369 (11)	0.0318 (9)	0.0054 (8)	0.0051 (7)	−0.0041 (8)
C16	0.0235 (9)	0.0243 (9)	0.0334 (9)	0.0075 (7)	−0.0026 (7)	−0.0058 (7)
C17	0.0297 (10)	0.0202 (9)	0.0276 (8)	0.0015 (7)	0.0011 (7)	0.0010 (7)
C18	0.0232 (9)	0.0193 (8)	0.0226 (8)	−0.0003 (6)	0.0045 (6)	−0.0008 (6)
C19	0.0281 (9)	0.0194 (8)	0.0198 (7)	−0.0040 (7)	0.0093 (6)	−0.0022 (6)
C20	0.0374 (11)	0.0218 (9)	0.0308 (9)	−0.0020 (8)	0.0109 (8)	0.0008 (7)
C21	0.0648 (15)	0.0209 (9)	0.0367 (10)	−0.0006 (9)	0.0198 (10)	0.0030 (8)
C22	0.0719 (16)	0.0245 (10)	0.0339 (10)	−0.0199 (10)	0.0271 (10)	−0.0073 (8)
C23	0.0464 (12)	0.0380 (11)	0.0286 (9)	−0.0224 (9)	0.0159 (8)	−0.0115 (8)
C24	0.0295 (9)	0.0308 (10)	0.0231 (8)	−0.0096 (8)	0.0099 (7)	−0.0050 (7)
C25	0.0141 (7)	0.0198 (8)	0.0218 (7)	0.0000 (6)	0.0035 (6)	−0.0002 (6)
C26	0.0177 (8)	0.0210 (9)	0.0286 (8)	0.0005 (6)	0.0047 (6)	−0.0003 (6)
C27	0.0177 (8)	0.0219 (9)	0.0395 (10)	−0.0013 (7)	0.0039 (7)	−0.0043 (7)
C28	0.0202 (9)	0.0338 (10)	0.0327 (9)	−0.0024 (7)	0.0061 (7)	−0.0119 (8)
C29	0.0237 (9)	0.0380 (11)	0.0227 (8)	−0.0008 (8)	0.0054 (7)	−0.0041 (7)
C30	0.0181 (8)	0.0256 (9)	0.0248 (8)	0.0005 (7)	0.0058 (6)	0.0001 (6)
C31	0.0199 (8)	0.0171 (8)	0.0216 (7)	−0.0008 (6)	0.0024 (6)	0.0002 (6)
C32	0.0221 (8)	0.0217 (9)	0.0274 (8)	0.0007 (7)	0.0077 (7)	0.0034 (6)
C33	0.0302 (9)	0.0241 (9)	0.0243 (8)	−0.0057 (7)	0.0066 (7)	0.0022 (7)
C34	0.0477 (12)	0.0200 (9)	0.0321 (9)	−0.0004 (8)	0.0112 (8)	0.0052 (7)
C35	0.0518 (13)	0.0247 (10)	0.0483 (12)	0.0136 (9)	0.0232 (10)	0.0101 (8)
C36	0.0371 (11)	0.0241 (9)	0.0385 (10)	0.0062 (8)	0.0188 (8)	0.0055 (7)
C38	0.0186 (8)	0.0187 (8)	0.0199 (7)	0.0017 (6)	0.0053 (6)	0.0015 (6)
C40	0.0203 (8)	0.0198 (8)	0.0180 (7)	−0.0016 (6)	0.0046 (6)	0.0018 (6)
C41	0.0247 (9)	0.0197 (8)	0.0240 (8)	0.0015 (7)	0.0085 (6)	0.0006 (6)
C42	0.0235 (9)	0.0267 (9)	0.0250 (8)	0.0026 (7)	0.0099 (7)	0.0027 (6)
C43	0.0226 (9)	0.0243 (9)	0.0237 (8)	−0.0029 (7)	0.0058 (6)	0.0039 (6)
C44	0.0305 (9)	0.0202 (9)	0.0266 (8)	−0.0048 (7)	0.0083 (7)	−0.0017 (6)
C45	0.0303 (9)	0.0214 (9)	0.0236 (8)	−0.0009 (7)	0.0104 (7)	−0.0030 (6)
C5	0.0212 (9)	0.0312 (10)	0.0255 (8)	0.0024 (7)	0.0059 (6)	−0.0008 (7)

### Geometric parameters (Å, º)

**Table:**

Cu1—P2	2.2602 (4)	C16—H16	0.9500
Cu1—P1	2.2671 (4)	C17—C18	1.395 (2)
Cu1—S1	2.3782 (4)	C17—H17	0.9500
Cu1—Cl1	2.4023 (4)	C18—H18	0.9500
O1—N1	1.227 (2)	C19—C24	1.392 (2)
N1—O2	1.230 (2)	C19—C20	1.396 (3)
N1—C43	1.471 (2)	C20—C21	1.391 (3)
S1—C38	1.7031 (16)	C20—H20	0.9500
P1—C1	1.8283 (16)	C21—C22	1.387 (3)
P1—C31	1.8296 (16)	C21—H21	0.9500
P1—C25	1.8362 (16)	C22—C23	1.375 (3)
C1—C5	1.391 (2)	C22—H22	0.9500
C1—C2	1.394 (2)	C23—C24	1.400 (3)
P2—C19	1.8241 (17)	C23—H23	0.9500
P2—C7	1.8258 (15)	C24—H24	0.9500
P2—C13	1.8278 (16)	C25—C30	1.389 (2)
N2—C38	1.324 (2)	C25—C26	1.403 (2)
N2—H2A	0.882 (15)	C26—C27	1.387 (2)
N2—H2B	0.882 (14)	C26—H26	0.9500
C2—C3	1.394 (2)	C27—C28	1.389 (3)
C2—H2	0.9500	C27—H27	0.9500
C3—C6	1.382 (3)	C28—C29	1.386 (3)
C3—H3	0.9500	C28—H28	0.9500
N3—C38	1.3578 (19)	C29—C30	1.393 (2)
N3—C40	1.403 (2)	C29—H29	0.9500
N3—H3A	0.874 (14)	C30—H30	0.9500
C4—C6	1.385 (3)	C31—C36	1.392 (2)
C4—C5	1.387 (2)	C31—C32	1.399 (2)
C4—H4	0.9500	C32—C33	1.388 (2)
C6—H6	0.9500	C32—H32	0.9500
C7—C12	1.393 (2)	C33—C34	1.380 (3)
C7—C8	1.396 (2)	C33—H33	0.9500
C8—C9	1.391 (2)	C34—C35	1.387 (3)
C8—H8	0.9500	C34—H34	0.9500
C9—C10	1.385 (2)	C35—C36	1.388 (3)
C9—H9	0.9500	C35—H35	0.9500
C10—C11	1.381 (2)	C36—H36	0.9500
C10—H10	0.9500	C40—C45	1.391 (2)
C11—C12	1.394 (2)	C40—C41	1.396 (2)
C11—H11	0.9500	C41—C42	1.384 (2)
C12—H12	0.9500	C41—H41	0.9500
C13—C18	1.386 (2)	C42—C43	1.380 (2)
C13—C14	1.400 (2)	C42—H42	0.9500
C14—C15	1.381 (3)	C43—C44	1.383 (2)
C14—H14	0.9500	C44—C45	1.386 (2)
C15—C16	1.387 (3)	C44—H44	0.9500
C15—H15	0.9500	C45—H45	0.9500
C16—C17	1.384 (3)	C5—H5	0.9500
			
P2—Cu1—P1	129.119 (16)	C24—C19—C20	119.30 (16)
P2—Cu1—S1	101.267 (15)	C24—C19—P2	124.80 (14)
P1—Cu1—S1	110.861 (15)	C20—C19—P2	115.88 (13)
P2—Cu1—Cl1	99.870 (15)	C21—C20—C19	120.67 (19)
P1—Cu1—Cl1	109.823 (16)	C21—C20—H20	119.7
S1—Cu1—Cl1	102.637 (15)	C19—C20—H20	119.7
O1—N1—O2	123.66 (15)	C22—C21—C20	119.5 (2)
O1—N1—C43	118.11 (15)	C22—C21—H21	120.3
O2—N1—C43	118.21 (15)	C20—C21—H21	120.3
C38—S1—Cu1	105.77 (6)	C23—C22—C21	120.47 (18)
C1—P1—C31	102.01 (7)	C23—C22—H22	119.8
C1—P1—C25	103.01 (7)	C21—C22—H22	119.8
C31—P1—C25	103.74 (7)	C22—C23—C24	120.35 (19)
C1—P1—Cu1	117.47 (5)	C22—C23—H23	119.8
C31—P1—Cu1	114.17 (5)	C24—C23—H23	119.8
C25—P1—Cu1	114.62 (5)	C19—C24—C23	119.72 (18)
C5—C1—C2	119.14 (15)	C19—C24—H24	120.1
C5—C1—P1	117.67 (12)	C23—C24—H24	120.1
C2—C1—P1	123.07 (12)	C30—C25—C26	119.18 (15)
C19—P2—C7	103.12 (7)	C30—C25—P1	123.38 (13)
C19—P2—C13	106.11 (8)	C26—C25—P1	117.34 (12)
C7—P2—C13	103.61 (7)	C27—C26—C25	120.28 (16)
C19—P2—Cu1	111.81 (5)	C27—C26—H26	119.9
C7—P2—Cu1	121.54 (5)	C25—C26—H26	119.9
C13—P2—Cu1	109.40 (5)	C26—C27—C28	120.18 (17)
C38—N2—H2A	119.8 (13)	C26—C27—H27	119.9
C38—N2—H2B	116.9 (13)	C28—C27—H27	119.9
H2A—N2—H2B	122.0 (18)	C29—C28—C27	119.78 (16)
C1—C2—C3	120.03 (16)	C29—C28—H28	120.1
C1—C2—H2	120.0	C27—C28—H28	120.1
C3—C2—H2	120.0	C28—C29—C30	120.32 (17)
C6—C3—C2	120.11 (17)	C28—C29—H29	119.8
C6—C3—H3	119.9	C30—C29—H29	119.8
C2—C3—H3	119.9	C25—C30—C29	120.23 (16)
C38—N3—C40	130.96 (14)	C25—C30—H30	119.9
C38—N3—H3A	112.4 (13)	C29—C30—H30	119.9
C40—N3—H3A	116.6 (13)	C36—C31—C32	118.63 (15)
C6—C4—C5	119.77 (17)	C36—C31—P1	123.13 (13)
C6—C4—H4	120.1	C32—C31—P1	118.24 (12)
C5—C4—H4	120.1	C33—C32—C31	120.59 (16)
C3—C6—C4	120.21 (16)	C33—C32—H32	119.7
C3—C6—H6	119.9	C31—C32—H32	119.7
C4—C6—H6	119.9	C34—C33—C32	120.21 (16)
C12—C7—C8	119.15 (14)	C34—C33—H33	119.9
C12—C7—P2	118.20 (12)	C32—C33—H33	119.9
C8—C7—P2	122.64 (12)	C33—C34—C35	119.76 (17)
C9—C8—C7	120.13 (16)	C33—C34—H34	120.1
C9—C8—H8	119.9	C35—C34—H34	120.1
C7—C8—H8	119.9	C34—C35—C36	120.32 (18)
C10—C9—C8	120.27 (16)	C34—C35—H35	119.8
C10—C9—H9	119.9	C36—C35—H35	119.8
C8—C9—H9	119.9	C35—C36—C31	120.48 (17)
C11—C10—C9	120.01 (15)	C35—C36—H36	119.8
C11—C10—H10	120.0	C31—C36—H36	119.8
C9—C10—H10	120.0	N2—C38—N3	114.81 (14)
C10—C11—C12	120.12 (16)	N2—C38—S1	121.44 (12)
C10—C11—H11	119.9	N3—C38—S1	123.72 (12)
C12—C11—H11	119.9	C45—C40—C41	119.48 (15)
C7—C12—C11	120.30 (15)	C45—C40—N3	124.38 (15)
C7—C12—H12	119.9	C41—C40—N3	115.95 (15)
C11—C12—H12	119.9	C42—C41—C40	120.65 (16)
C18—C13—C14	118.77 (15)	C42—C41—H41	119.7
C18—C13—P2	122.95 (12)	C40—C41—H41	119.7
C14—C13—P2	117.94 (12)	C43—C42—C41	118.56 (15)
C15—C14—C13	120.59 (16)	C43—C42—H42	120.7
C15—C14—H14	119.7	C41—C42—H42	120.7
C13—C14—H14	119.7	C42—C43—C44	122.06 (16)
C14—C15—C16	120.25 (16)	C42—C43—N1	118.82 (15)
C14—C15—H15	119.9	C44—C43—N1	119.09 (16)
C16—C15—H15	119.9	C43—C44—C45	118.96 (16)
C17—C16—C15	119.76 (16)	C43—C44—H44	120.5
C17—C16—H16	120.1	C45—C44—H44	120.5
C15—C16—H16	120.1	C44—C45—C40	120.24 (15)
C16—C17—C18	120.02 (16)	C44—C45—H45	119.9
C16—C17—H17	120.0	C40—C45—H45	119.9
C18—C17—H17	120.0	C4—C5—C1	120.72 (16)
C13—C18—C17	120.58 (16)	C4—C5—H5	119.6
C13—C18—H18	119.7	C1—C5—H5	119.6
C17—C18—H18	119.7		
			
C31—P1—C1—C5	88.90 (14)	C22—C23—C24—C19	−1.0 (3)
C25—P1—C1—C5	−163.75 (13)	C1—P1—C25—C30	−107.34 (14)
Cu1—P1—C1—C5	−36.73 (15)	C31—P1—C25—C30	−1.30 (16)
C31—P1—C1—C2	−87.15 (15)	Cu1—P1—C25—C30	123.85 (13)
C25—P1—C1—C2	20.21 (16)	C1—P1—C25—C26	76.16 (13)
Cu1—P1—C1—C2	147.23 (12)	C31—P1—C25—C26	−177.80 (12)
C5—C1—C2—C3	0.3 (3)	Cu1—P1—C25—C26	−52.65 (13)
P1—C1—C2—C3	176.27 (13)	C30—C25—C26—C27	−0.6 (2)
C1—C2—C3—C6	−0.6 (3)	P1—C25—C26—C27	176.02 (13)
C2—C3—C6—C4	0.1 (3)	C25—C26—C27—C28	−0.6 (2)
C5—C4—C6—C3	0.6 (3)	C26—C27—C28—C29	1.2 (3)
C19—P2—C7—C12	−163.25 (13)	C27—C28—C29—C30	−0.5 (3)
C13—P2—C7—C12	86.29 (14)	C26—C25—C30—C29	1.3 (2)
Cu1—P2—C7—C12	−37.05 (15)	P1—C25—C30—C29	−175.13 (13)
C19—P2—C7—C8	15.84 (15)	C28—C29—C30—C25	−0.8 (3)
C13—P2—C7—C8	−94.63 (14)	C1—P1—C31—C36	3.32 (17)
Cu1—P2—C7—C8	142.04 (12)	C25—P1—C31—C36	−103.47 (15)
C12—C7—C8—C9	0.6 (2)	Cu1—P1—C31—C36	131.09 (14)
P2—C7—C8—C9	−178.45 (13)	C1—P1—C31—C32	−176.22 (13)
C7—C8—C9—C10	−1.1 (3)	C25—P1—C31—C32	76.99 (14)
C8—C9—C10—C11	0.3 (3)	Cu1—P1—C31—C32	−48.45 (14)
C9—C10—C11—C12	1.1 (3)	C36—C31—C32—C33	1.1 (3)
C8—C7—C12—C11	0.7 (2)	P1—C31—C32—C33	−179.30 (13)
P2—C7—C12—C11	179.84 (13)	C31—C32—C33—C34	−1.1 (3)
C10—C11—C12—C7	−1.6 (3)	C32—C33—C34—C35	0.3 (3)
C19—P2—C13—C18	−127.51 (14)	C33—C34—C35—C36	0.3 (3)
C7—P2—C13—C18	−19.27 (15)	C34—C35—C36—C31	−0.3 (3)
Cu1—P2—C13—C18	111.71 (13)	C32—C31—C36—C35	−0.5 (3)
C19—P2—C13—C14	59.38 (14)	P1—C31—C36—C35	179.99 (16)
C7—P2—C13—C14	167.63 (13)	C40—N3—C38—N2	−170.95 (16)
Cu1—P2—C13—C14	−61.39 (14)	C40—N3—C38—S1	10.9 (3)
C18—C13—C14—C15	2.1 (3)	Cu1—S1—C38—N2	6.43 (15)
P2—C13—C14—C15	175.53 (14)	Cu1—S1—C38—N3	−175.53 (12)
C13—C14—C15—C16	−1.8 (3)	C38—N3—C40—C45	29.7 (3)
C14—C15—C16—C17	0.3 (3)	C38—N3—C40—C41	−155.33 (17)
C15—C16—C17—C18	0.7 (3)	C45—C40—C41—C42	2.5 (3)
C14—C13—C18—C17	−1.1 (2)	N3—C40—C41—C42	−172.74 (15)
P2—C13—C18—C17	−174.11 (13)	C40—C41—C42—C43	−1.6 (3)
C16—C17—C18—C13	−0.3 (3)	C41—C42—C43—C44	−0.2 (3)
C7—P2—C19—C24	−92.07 (15)	C41—C42—C43—N1	177.84 (15)
C13—P2—C19—C24	16.52 (16)	O1—N1—C43—C42	−172.69 (16)
Cu1—P2—C19—C24	135.72 (13)	O2—N1—C43—C42	5.9 (2)
C7—P2—C19—C20	86.40 (13)	O1—N1—C43—C44	5.4 (2)
C13—P2—C19—C20	−165.01 (12)	O2—N1—C43—C44	−176.00 (16)
Cu1—P2—C19—C20	−45.80 (14)	C42—C43—C44—C45	1.0 (3)
C24—C19—C20—C21	0.8 (3)	N1—C43—C44—C45	−177.05 (15)
P2—C19—C20—C21	−177.76 (14)	C43—C44—C45—C40	0.0 (3)
C19—C20—C21—C22	−1.5 (3)	C41—C40—C45—C44	−1.7 (3)
C20—C21—C22—C23	1.0 (3)	N3—C40—C45—C44	173.11 (16)
C21—C22—C23—C24	0.2 (3)	C6—C4—C5—C1	−0.8 (3)
C20—C19—C24—C23	0.4 (2)	C2—C1—C5—C4	0.4 (3)
P2—C19—C24—C23	178.87 (13)	P1—C1—C5—C4	−175.79 (14)

### Hydrogen-bond geometry (Å, º)

**Table:**

*D*—H···*A*	*D*—H	H···*A*	*D*···*A*	*D*—H···*A*
N2—H2*A*···Cl1^i^	0.88 (2)	2.35 (2)	3.1974 (14)	160 (2)
N2—H2*B*···Cl1	0.88 (1)	2.42 (2)	3.2504 (15)	158 (2)
N3—H3*A*···Cl1^i^	0.87 (1)	2.49 (2)	3.3199 (14)	158 (2)
C9—H9···O1^ii^	0.95	2.57	3.303 (2)	135
C30—H30···O2^iii^	0.95	2.70	3.386 (2)	130

Symmetry codes: (i) *x*, −*y*+1/2, *z*+1/2; (ii) −*x*, −*y*, −*z*+1; (iii) *x*+1, *y*, *z*.
